# Maturation of the Pupil Light Reflex Occurs Until Adulthood in Mice

**DOI:** 10.3389/fneur.2019.00056

**Published:** 2019-02-04

**Authors:** Noémie Kircher, Sylvain V. Crippa, Catherine Martin, Aki Kawasaki, Corinne Kostic

**Affiliations:** ^1^Group for Retinal Disorder Research, Department of Ophthalmology, Hôpital Ophtalmique Jules Gonin, University of Lausanne, Lausanne, Switzerland; ^2^Neuro-Ophtalmology, Department of Ophthalmology, Hôpital Ophtalmique Jules Gonin, University of Lausanne, Lausanne, Switzerland

**Keywords:** pupil light response, electroretinography, C57BL6, Sv129S6, age-related changes, retina, maturation

## Abstract

With respect to photoreceptor function, it is well known that electroretinogram (ERG) amplitudes decrease with age, but to our knowledge, studies describing age-related changes in the pupil light response (PLR) of mice are lacking. This study recorded the PLR and ERG in C57BL/6 and Sv129S6 wild-type mice at three different ages during early adulthood. Dark- and light-adapted PLR and ERG measurements were performed at 1, 2, and 4 months of age. For PLR measurements, we used either a red (622 nm) or blue (463 nm) light stimulus (500 ms) to stimulate one eye. We selected various light intensities ranging across almost 4 log units and subsequently classified them as low, medium, or high intensity. From the recorded PLR, we selected parameters to quantify the early and late phases of the response such as the baseline pupil size, the maximal constriction amplitude, the maximal velocity, the early partial dilation (area under the curve of the positive peak of the first derivative of PLR tracing), and the sustained constriction amplitude. For ERG measurements, both scotopic and photopic responses were recorded following stimulation with green light (520 nm) at preselected intensities. The amplitudes and latencies of the a-wave and the b-wave were also analyzed. In both strains, 1-month-old animals presented with a smaller baseline pupil diameter compared to that in 2- and 4-month-old mice. They also exhibited greater maximal constriction amplitude in response to red stimuli of medium intensity. Further, 1-month-old Sv129S6 mice responded with greater constriction amplitude to all other red and blue stimuli. One-month-old C57BL/6 mice also demonstrated faster early partial dilation and smaller sustained response to low blue stimuli. The ERG of 1-month-old C57BL/6 mice showed a greater scotopic a-wave amplitude compared to that of 2-month-old mice, whereas no significant differences were found in Sv129S6 mice. These results suggest that the functional maturation of the neuronal pathway that mediates the PLR continues after 1 month of age. In studies that measure PLR to determine retinal integrity in adult mice, it is thus important to determine normative values in animals of 2 months of age.

## Introduction

The electroretinogram is a standardized test to described outer photoreceptor function, and normative values for rod and cone activity have been established in adult humans (1, 2). This technique has the advantage of recording the electrical activity of photoreceptors and interneurons, eliminating potential post-retinal effects. However, when vision is severely affected and reaches the level of light perception, the full-field electroretinogram (ERG) response becomes undetectable, as observed in patients affected by retinitis pigmentosa (3–7).

Alternatively, the pupillary light response (PLR) can provide a functional evaluation of outer and inner photoreceptors (7, 8). By modifying the wavelength, intensity, and background conditions of the light stimulus, the specific contribution of different photoreceptors to the PLR can be altered to favor rods, cones, or intrinsically photosensitive retinal ganglion cells (ipRGCs) (6, 9–12). However, a standardized protocol and normative values to assess the PLR in adult humans has not been defined.

In a previous study, we developed a PLR protocol for mice to characterize changes in the pupil response that are related to rod and cone degenerative diseases. When *Rho*^−/−^ (rodless) mice were exposed to blue- and red-light stimuli, the initial maximal constriction amplitude was decreased, whereas the response after light termination (sustained constriction amplitude) was increased compared to that in wild-type mice. These findings implied that rod photoreceptors are a major contributor to both initial and post-illumination pupil constriction. Furthermore, low- or medium-intensity red light was not able to elicit any pupil response in *Cnga3*^−/−^*; Rho*^−/−^ (coneless and rodless) mice demonstrating that both rods and cones are required to promote pupil responses under these particular conditions (13). Many other studies, each using different methodologies, have examined the origin of the photosensitive input with respect to the murine PLR. In addition to rods and cones, a small subset of retinal ganglion cells expressing the melanopsin protein, termed ipRGCs, was found to contribute to the mouse PLR (14–18). The general model is that rods are required for PLR sensitivity to lower intensity stimuli, whereas cones and melanopsin cells induce responses to more intense levels of light (16, 17). Moreover, rods and cones are mainly involved in the rapid and transient pupil response, whereas ipRGCs are the predominant players in the sustained pupillary response (17, 18).

ERG recordings change with age in young rodents. Specifically, the response amplitudes recorded from photoreceptors and second-order neurons increase gradually from eye-opening (postnatal day 12, P12) until adulthood, which is approximately P30 in mice (19). In rats, oscillatory potential (OP) amplitude and the implicit time also change with age. The amplitudes of OP2, OP3, and OP4 are larger at P31 than at P18 and P67, suggesting the functional refinement of the inner retina (20). Moreover, between 1 and 2 months of age, the mixed rod–cone response and the photopic cone response decrease (21). These observations suggest that the development of retinal processing continues after the first 2 postnatal weeks.

The development of the mouse retina starts at the embryonic stage and continues after birth. Retinal ganglion cells (RGCs) differentiate first followed by amacrine, cone, and horizontal cells. However, the neurogenesis of rods and bipolar cells continues for 1–2 weeks after birth (22). The process that converts bistratified ON-OFF responsive RGCs to monostratified ON or OFF responsive RGCs occurs 2–3 weeks after eye-opening (P12) and depends on light exposure (23). The synaptic strength, measured as the frequency of spontaneous synaptic inputs, also continues to mature after P12, and RGC spontaneous activities peak at P25, finally decreasing to reach adult levels at P60 (23). OFF-type bipolar cells, for example, those responsive to decreases in light, retain the ability to form new synapses in the intact adult retina and continue to increase synapse numbers and the complexity of dendritic arborization to at least 6 months of age, well after the mouse retina is considered mature (24). It has been shown that the presence of abnormal synaptic ribbons (synaptic ribbons floating in the cytoplasm without post-synaptic processes) correlates with abnormal ERG responses (reduction in the amplitude and increase in the implicit time of the b-wave) (25). Although impaired synaptogenesis has an effect on ERG measurements, the influence of normally developing synaptogenesis on the ERG remains unclear.

Five subtypes of ipRGCs (1–3% of the RGC population) were described in rodents based on the stratification of their dendrites (26). McNeil et al. (27) showed that ipRGC neurogenesis begins from embryonic day (ED) 11 to ED14, similar to that observed for other RGCs, but continues after ED15 when other types of RGC neurogenesis stop. At ED15, ipRGCs are not present in the peripheral retina and reach the ciliary margin at birth (P0). IpRGCs begin innervating the suprachiasmatic nucleus at P3 and P4 until the second postnatal week, whereas most RGCs innervate their image-forming targets during embryogenesis. Moreover, the appearance of ipRGC axons in the olivary pretectal nucleus coincides precisely with the onset of the PLR at P7 (27). However, the consequence of retina circuitry refinement, observed after eye opening on visual function is still not well understood.

In this study, we examined the effect of continued retinal maturation after 1 month of age, based on functional tests of the retina, in mice. Specifically, we aimed to better characterize how age affects the mouse PLR and ERG response under non-pathological conditions. Two wild-type mouse strains, C57BL/6 and Sv129S6, were examined because many retinal dystrophy mouse models are based on these genetic backgrounds. Additionally, a difference in the course of degeneration was observed when comparing *Rho*^−/−^ mice between C57BL/6 and Sv129S6 backgrounds (28). The development of such normative values will help to differentiate pathological responses from non-relevant variations, when assessing functional retinal integrity.

## Materials and Methods

### Animals

Animals were handled in accordance with the statement of the “Animals in Research Committee” of the Association for Research in Vision and Ophthalmology, and protocols were approved by the local institutional committee (VD1367). The mice were maintained at 22°C with a 12-h light/12-h dark cycle with light on at 7:00 a.m. and were feed *ad libitum*. C57BL/6 wild-type (males, *n* = 15; females, *n* = 12) and Sv129S6 wild-type (males, *n* = 7; females, *n* = 11) mice were tested at 1, 2, and 4 months. Dark- and light-adapted PLR and ERG examination were always performed on separate days and during the morning, specifically during the first 6 h of the light cycle, at week 4 (1 month), week 8 (2 months), and week 16 (4 months). ERG examinations were always performed after the light and dark-adapted PLR to avoid the effects of the anesthesia on the PLR.

### Light Stimuli and Pupil Response Recording

Mice were dark-adapted overnight and tested under mesopic (< 5 lux) red light. Pupillary recordings were performed as previously described (13). Animals were not anesthetized to avoid the effects of medication, but were manually restrained in front of the camera. Pupils were maintained at a constant distance from the camera of the A2000 pupillometer (Neuroptics Inc., Irvine, CA). This apparatus presents a light stimulus to one eye while continuously recording the pupil diameter at 31 Hz in the same eye. For this study, the light stimulus had a duration of 500 ms and was either red (622 nm) or blue (463 nm), both with a half-maximum bandwidth of 8 nm, with a range of intensities covering almost 4 log units. Light is emitted through a diffusing screen (approximately 50° × 35° of the visual angle). Based on a previous study, we used pre-selected light intensities that were considered “low,” “medium,” and “high” [(13); Table 1]. Low light intensities are sufficient to generate more than 10% constriction. The maximum red intensity was determined based on the limit of the pupillometer apparatus, whereas the maximum blue intensity was limited to restrict the response to less than 50% of the constriction amplitude in an effort to minimize mouse discomfort. We used the following light stimulus sequence to test all animals under scotopic or photopic conditions: low red, low blue, medium red, medium blue, high red, high blue (Table 1). We recorded the pupil response once after administering each stimulus in this sequence for any given animal of a particular age.

**Table 1:** Light stimulus intensities converted to different units and the order of stimuli applied during the protocol of this study.

**Stimuli name**	**Intensity**		
	**(log cds/m^**2**^)**	**(log W/m^**2**^)**	**(W/m^**2**^)**
Low red	1.2	−1.2	0.065
Low blue	0.6	−1.1	0.074
Medium red	2	−0.4	0.408
Medium blue	1.2	−0.5	0.3
High red	4.5	2.1	129.018
High blue	2	0.3	1.893

The pupil recording started 500 ms before administering the light stimulus and continued 29 s after the blue light stimulus offset or 17 s after the red-light stimulus offset. The interval between stimuli was at least 49 s after blue-light stimulations or 37 s after red-light stimulations; this provided the opportunity for the mouse to freely move for at least 20 s after recording each stimulus to calm the animals before the next stimulus.

The PLR recordings under photopic conditions were taken in an independent session 1 day before or after the dark-adapted recordings. Mice were exposed to room light (fluorescent tube white light emitting 200 ± 50 lux at the level of the mice) for 30 min before the test and both eyes were constantly exposed to the same ambient light during pupil response recordings for the stimulated eye.

### PLR Analysis

The raw data were exported to a worksheet and all pupil diameters were converted to a percentage of the baseline diameter. The following parameters were determined from the data.

The baseline pupil diameter was set as the mean pupil diameter during the 500 ms before light onset; thereafter, all pupil sizes were converted to a relative size that was a function of the baseline value.

The pupil response was then divided into the constriction phase (defined as the time from the light onset to 2 s after light onset) and the recovery phase (defined as the time from the maximal constriction amplitude to the end of the recording at 29 or 17 s after blue or red stimuli offset, respectively). To evaluate the constriction phase, we determined the maximal constriction amplitude and the maximal velocity (see below). The recovery phase was analyzed at two different stages as follows: at an early phase, to determine the early partial dilation, until 2.5 s after light onset (first derivative, as follows) and at a later time point 9.5 s after light offset (sustained constriction amplitude and ratio, as follows). These features are more precisely described in the following text and in the formula listed in Table 2.

**Table 2:** Formulas related to pupil light response (PLR) parameters used for quantification.

Baseline pupil diameter	Sum of the pupil diameters (mm) during 500 ms before the light stimulus/total number of pupil diameter values during 500 ms before the light stimulus.
Maximal constriction amplitude	Baseline pupil diameter—minimal pupil diameter expressed in percentage.
Area under the curve	The first derivative of individual pupil tracing was created using GraphPad Prism 5.01 software (GraphPad Software, Inc., San Diego, CA, USA). Individual curves were then exported into excel for identification of positif peak by the formula: = SI(ET(Cn>0;Cn+1>0);($An-$An-1)*(Cn+Cn+1)/2;“”) where C is the column of data for one individual, A is the colunm for time values, n is the row number. The AUC of each individual is then determined by the sum of these values.
Sustained response	Percentage of constriction amplitude at 9.5 s following stimulus offset.
Ratio of the sustained response	Percentage of constriction amplitude at 9.5 s following stimulus offset above the maximal constriction amplitude.

The maximal constriction amplitude was the percent change from the baseline value to the minimal diameter reached after application of the light stimuli during the first 2 s following stimulus onset. For the photopic protocol, instead of using the maximal constriction amplitude, we used the minimal diameter reached during the first 1.8 s following stimulus onset, which is the absolute value of the diameter (mm) at the maximal constriction point.

To characterize the early response dynamics by determining the maximal velocity (constriction phase) and the early partial dilation (early recovery phase), the first derivative curve was created using GraphPad Prism 5.01 software (GraphPad Software, Inc., San Diego, CA, USA) comprising the first 3 s of the protocol (2.5 s from light onset). When the pupil was in a steady state, for example before the stimulus onset, the derivative values were essentially zero. A change resulting in a smaller pupil size was indicated by negative values, and the rate of change was indicated by the magnitude of these negative values. The peak negative value thus represented the maximum velocity of constriction, which becomes slower as constriction continues toward the maximum amplitude. When constriction stabilized briefly at the minimum pupil size, the derivative value returned to zero. Thereafter, if the derivative values became positive, this indicated a change resulting in an increase in pupil size, which corresponds to a dilation movement. When these positive values increased rapidly, a peak was distinguishable, indicating a rapid and early dilation for which the maximal velocity (the positive peak) was reached within the first 3 s of the recording. Alternatively, if the derivative was maintained at approximately zero, without reaching a peak positive value, this indicated the absence of a rapid dilation within this 3 s period. To quantify this early partial dilation, we calculated the area under the curve (AUC) below this peak, when the first derivative was positive.

The sustained constriction amplitude was the percent change from the baseline value to the diameter reached at 9.5 s following stimulus offset. In the recovery phase, this time point was previously demonstrated to reveal the most significant difference between the different photoreceptor cell input conditions (13).

To evaluate the relative recovery from the maximal constriction, we calculated the ratio of the sustained constriction amplitude to the maximal constriction amplitude.

### Electroretinography

Mice were dark-adapted overnight for another session of ERG measurements 1 or 2 days after pupillometry. They were anesthetized with a mixture of ketamine (20 mg/kg, Streuli, Uznach, CH) and xylazine (20 mg/kg, Bayler, Lyssach, CH) and both pupils were dilated with a single eye drop of 0.5% tropicamide (Théa, Schaffausen, CH) and 5% phenylephrine hydrochloride (Bausch and Lomb, London, UK). As mice are temperature-sensitive, animals were maintained on a heating pad connected to a temperature control unit to maintain temperature at 37–38°C throughout the experiment. Responses to standard single light flashes [520 nm; half-bandwidth, 35 nm; at 0.0001 cds/m^2^ (2.2 × 10^−7^ W/sr/m^2^), 0.001 cds/m^2^ (2.2 × 10^−6^ W/sr/m^2^), 0.01 cds/m^2^ (2.2 × 10^−5^ W/sr/m^2^), 0.03 (6.6 × 10^−5^ W/sr/m^2^), 0.1 cds/m^2^ (2.2 × 10^−4^ W/sr/m^2^), 0.3 cds/m^2^ (6.6 × 10^−4^ W/sr/m^2^), 1 cds/m^2^ (2.2 × 10^−3^ W/sr/m^2^), 3 cds/m^2^ (6.6 × 10^−3^ W/sr/m^2^), 10 cds/m^2^ (1.9 × 10^−2^ W/sr/m^2^), and 30 cds/m^2^ (5.9 × 10^−2^ W/sr/m^2^) for scotopic ERG and 1 cds/m^2^ (2.2 × 10^−3^ W/sr/m^2^), 3 cds/m^2^ (6.6 × 10^−3^ W/sr/m^2^), 10 cds/m^2^ (1.9 × 10^−2^ W/sr/m^2^), and 30 cds/m^2^ (5.9 × 10^−2^ W/sr/m^2^) for photopic ERG] generated by a stroboscope (Ganzfeld stimulator, Espion E3 apparatus; Diagnosys LLC, Lowell, MA, USA) were recorded binocularly with corneal electrodes. The a-wave (photoreceptor-driven first negative wave) amplitude was measured from baseline to the bottom of the a-wave trough and the b-wave (second order neuron-driven, first positive wave) amplitude was measured from the bottom of the a-wave trough to the b-wave peak.

### Immunohistochemistry

Three eyes from three different C57BL/6 mice, 1 and 2 months of age, were prepared for immunohistochemistry. A cauterization mark was made in the inner corner of the eye as a marker for orientation. After enucleation, eyes were incubated at room temperature (RT) in 4% paraformaldehyde for 1.25 h, washed twice with PBS, and incubated sequentially for 2 h each in 10% and 20% sucrose and finally overnight in 30% sucrose. Eyes were embedded in yazzulla (30% egg albumin and 3% gelatin in water) and cut with a cryostat to generate 14-μm-thick sections.

For all staining procedures, blocking was performed at RT for 1–1.5 h, and the primary antibody was incubated with the samples at 4°C overnight, whereas the secondary antibody was added to the section for 1 h at RT. For protein kinase C-alpha (PKC) and bassoon double staining, sections were blocked with 5% normal goat serum with 0.2% triton X-100, and anti-PKC-alpha (sc-10800, Santa Cruz, Dallas, USA) and anti-Bassoon (VAM-PS003, Stressgen, Lausen, Switzerland) were diluted to 1:200 and 1:400, respectively. For calbindin and cholinergic amacrine cell (ChAT) double staining, blocking was performed with 10% NDS with 0.2% triton X-100 before incubation with anti-Calbindin (1:5000, SWANT CB 38, Swant Inc., Marly, Switzerland) and anti-ChAT (1:2500, kind gift from Prof. J.P Hornung, UNIL, Lausanne, Switzerland) antibodies. The blocking solution for transducin Gαt1 rods (GNAT1) was 10% NGS with 0.3% triton X-100 in PBS and anti-GNAT1 antibody was diluted to 1:1000 (Sc-389 Santa Cruz, Dallas, USA). S-opsin/MWL-opsin double staining was performed by first blocking with 5% normal donkey serum with 0.2% triton X-100 and anti-S-opsin (sc-14363, Santa Cruz, Dallas) and anti-MWL-Opsin (AB5405, Chemicon, Temecula, CA, USA) were diluted to 1:1000. Secondary antibodies including Alexa Fluor 488, 633, or 594 goat anti-rabbit, goat anti-mouse, donkey anti-goat, or donkey anti-rabbit antibodies (depending on the primary antibody) were diluted to 1:2000 in PBS and counterstaining was finally performed with DAPI. Sections were mounted in Mowiol® 4-88 reagent (Sigma, Buchs, Switzerland), a poly(vinyl)alcohol medium used to preserved stained sections.

### Immunohistochemistry Analysis

Images of the different labeled samples were obtained in 3D using LAST X software driving a DM6 Leica microscope and were merged to obtain a composite picture. Noise was removed by performing deconvolution using Huygens Essential software (Scientific Volume Imaging B.V. Hilversum, The Netherlands).

Quantification of the number of labeled cells was performed in the central section bisecting the optic nerve along the vertical axis, which represents the most sagittal region of the retina. The number of positive cells, based on different markers, was determined for the inferior and the superior hemispheres through manual counting during visualization using a microscope. Both inferior and superior counts were added together to obtain the final number of labeled cells in the entire section. Three eyes from three different mice were analyzed for each age group.

### Statistical Analysis

All statistical analyses were performed using GraphPad Prism software (San Diego, CA, USA). For the statistical analysis, each PLR characteristic obtained for each age group was analyzed by performing a two-way ANOVA with Bonferroni tests to compare males and females. As no sex-based difference was noted, males and females were then grouped as a single experimental group for each age tested. Two-way ANOVA and Bonferroni tests were then performed for each feature to compare the three C57BL/6 age groups or the two Sv129S6 age groups.

For immunochemistry, *t*-tests were performed for each marker to compare counts between 1 and 2 months of age. A value was considered significant if *p* < 0.05.

## Results

### Age-Related Changes in Baseline Pupil Diameter

Under scotopic conditions, the pupil baseline diameter increased significantly with age in both C57BL/6 (Table 3; 55% increase at 2 months and 74% increase at 4 months compared to that at 1 month; *p* < 0.001 for 1 vs. 2 and 4 months) and Sv126S6 strains (Table 4; 22% increase at 2 months compared to that at 1 month; *p* < 0.001). We also observed significantly larger pupil diameters in 2-month-old mice compared to those in 1-month-old C57BL/6 mice under photopic conditions (Figure 1A). Four-month-old C57BL/6 mice and Sv129S6 mice were not tested under photopic conditions. The smaller pupil diameter of 1-month-old mice consequently resulted in a reduction in the pupil area through which the light stimuli can enter. For the C57BL/6 strain, in dark-adapted conditions, a 60% reduction in pupil area was estimated compared to that in 2-month-old mice and a 77% reduction was calculated compared to that in 4-month-old animals. In the dark-adapted Sv126S6 strain, 1-month-old mice presented with a 33% reduced pupil area compared to that in 2-month-old animals. Since in the following experiments, the stimulus light intensities were kept constant, the amount of light entering the eye would proportionally decrease in 1-month-old mice.

**Table 3:** Pupil light response (PLR) analysis of C57BL/6 mice at 1, 2, and 4 months of age.

	**1 month**			**2 months**			**4 months**		
	**Mean**	**SEM**	***n***	**Mean**	**SEM**	***n***	**Mean**	**SEM**	***n***
**BASELINE PUPIL DIAMETER (mm)**									
	1.11	0.07	16	1.74^*^	0.08	15	1.93^*^	0.05	12
**MAXIMUM CONSTRICTION AMPLITUDE (%)**									
Low blue	36.27	1.02	23	36.38	1.34	13	35.09	1.58	13
Medium blue	43.86	1.32	23	45.43	2.45	14	45.07	1.49	10
High blue	52.41	1.87	16	53.10	1.82	16	49.84	3.54	8
Low red	20.19	1.26	22	15.35	1.14	17	18.19	2.14	14
Medium red	32.61	1.32	24	27.15^#^	0.86	16	25.29^†^	1.36	11
High red	44.96	1.80	20	43.79	1.74	16	41.22	2.21	12
**MAXIMAL CONSTRICTION VELOCITY (mm/s)**									
Low blue	−0.71	0.04	17	−0.87	0.06	13	−0.98^†^	0.04	13
Medium blue	−0.76	0.04	24	−0.95^#^	0.05	14	−1.05^*^	0.04	10
High blue	−0.84	0.06	16	−1.15^*^	0.09	15	−0.95	0.06	8
Low red	−0.46	0.05	22	−0.51	0.05	17	−0.59	0.06	14
Medium red	−0.73	0.05	24	−0.79	0.04	17	−0.79	0.05	12
High red	−0.82	0.05	20	−1.03^†^	0.06	16	−1.05^#^	0.06	12
**AREA UNDER THE CURVE OF THE POSITIVE PEAK OF THE 1ST DERIVATIVE**									
Low blue	9.63	1.11	24	5.78^†^	0.93	13	6.28^#^	1.35	13
Medium blue	2.91	0.89	24	1.34	0.49	14	1.27	1.06	10
High blue	0.39	0.28	17	0.13	0.086	15	0.11	0.08	8
Low red	8.67	1.02	21	6.13	0.62	16	7.46	0.67	14
Medium red	10.07	0.98	25	8.37	0.74	16	8.98	1.04	12
High red	2.79	0.55	27	2.28	0.44	15	1.64	0.49	12
**SUSTAINED CONSTRICTION AMPLITUDE (%)**									
Low blue	14.08	1.80	23	17.49	2.23	13	13.09	1.35	13
Medium blue	19.92	2.41	23	28.22^#^	1.84	14	25.63	1.83	10
High blue	32.25	2.98	17	45.51^*^	3.55	16	31.72	4.00	8
Low red	11.95	4.38	22	6.57	1.32	17	4.12	0.82	14
Medium red	9.16	1.73	24	16.81	5.33	17	9.24	1.17	12
High red	22.36	1.73	20	25.94	2.36	16	19.35	2.00	12
**RATIO**									
Low blue	0.38	0.04	23	0.48	0.06	13	0.38	0.04	13
Medium blue	0.46	0.05	25	0.64^#^	0.05	14	0.57	0.04	10
High blue	0.65	0.05	16	0.84^#^	0.06	15	0.64	0.07	8
Low red	0.46	0.08	21	0.41	0.08	17	0.27	0.06	14
Medium red	0.29	0.05	23	0.45	0.05	16	0.37	0.06	12
High red	0.50	0.03	20	0.59	0.04	16	0.47	0.04	12

*Quantification of the baseline pupil diameter (mm), the relative maximal constriction amplitude (%), the maximal constriction velocity (mm/s), the area under the curve of the positive peak of the first derivative (arbitrary unit), the relative sustained constriction amplitude (%), and the ratio of sustained to maximal constriction amplitude*.

* *Significantly different compared to 1-month-old mice, p < 0.001*;

† *significantly different compared to 1-month-old animals, p < 0.01*;

# *significantly different compared to 1-month-old mice, p < 0.05*.

**Table 4:** Pupil light response (PLR) analysis of Sv129S6 mice at 1 and 2 months of age.

	**1 month**			**2 months**		
	**Mean**	**SEM**	***n***	**Mean**	**SEM**	***n***
**BASELINE PUPIL DIAMETER (mm)**						
	1.30	0.04	17	1.58^*^	0.04	25
**MAXIMAL CONSTRICTION AMPLITUDE (%)**						
Low blue	42.87	1.007	24	36.74^#^	1.09	24
Medium blue	46.99	1.67	21	41.63^#^	1.34	24
High blue	50.91	1.68	22	43.75^†^	2.28	23
Low red	32.51	1.70	22	24.32^*^	1.29	25
Medium red	42.95	1.45	21	33.84^*^	0.97	25
High red	48.29	1.50	20	41.60^†^	1.49	26
**AREA UNDER THE CURVE OF THE POSITIVE PEAK OF THE 1ST DERIVATIVE**						
Low blue	15.71	1.17	24	14.07	0.71	24
Medium blue	7.94	1.11	20	6.99	0.82	27
High blue	2.27	0.77	20	1.94	0.61	25
Low red	17.19	1.13	21	12.67^†^	0.82	28
Medium red	20.56	1.23	24	15.95^†^	0.66	29
High red	9.60	1.00	20	9.19	0.92	32
**SUSTAINED CONSTRICTION AMPLITUDE (%)**						
Low blue	8.38	1.33	24	9.50	1.32	24
Medium blue	13.74	1.58	21	15.51	2.19	24
High blue	23.77	2.07	21	18.82	1.84	23
Low red	9.71	4.42	22	6.48	0.66	25
Medium red	7.03	1.09	22	8.11	1.09	25
High red	10.39	1.32	20	17.04	3.47	26
**RATIO**						
Low blue	0.17	0.03	24	0.22	0.03	24
Medium blue	0.26	0.04	21	0.30	0.03	24
High blue	0.41	0.03	21	0.38	0.03	23
Low red	0.34	0.22	22	0.21	0.03	25
Medium red	0.13	0.02	22	0.20	0.03	25
High red	0.18	0.02	20	0.37	0.07	26

*Quantification of the baseline pupil diameter (mm), the relative maximal constriction amplitude (%), the area under the curve of the positive peak of the first derivative (arbitrary unit), the relative sustained constriction amplitude (%), and the ratio of sustained to maximal constriction amplitude*.

* *Significantly different compared to 1-month-old mice, p < 0.001*;

† *significantly different compared to 1-month-old animals, p < 0.01*;

# *significantly different compared to 1-month-old mice, p < 0.05*.

**Figure 1:**
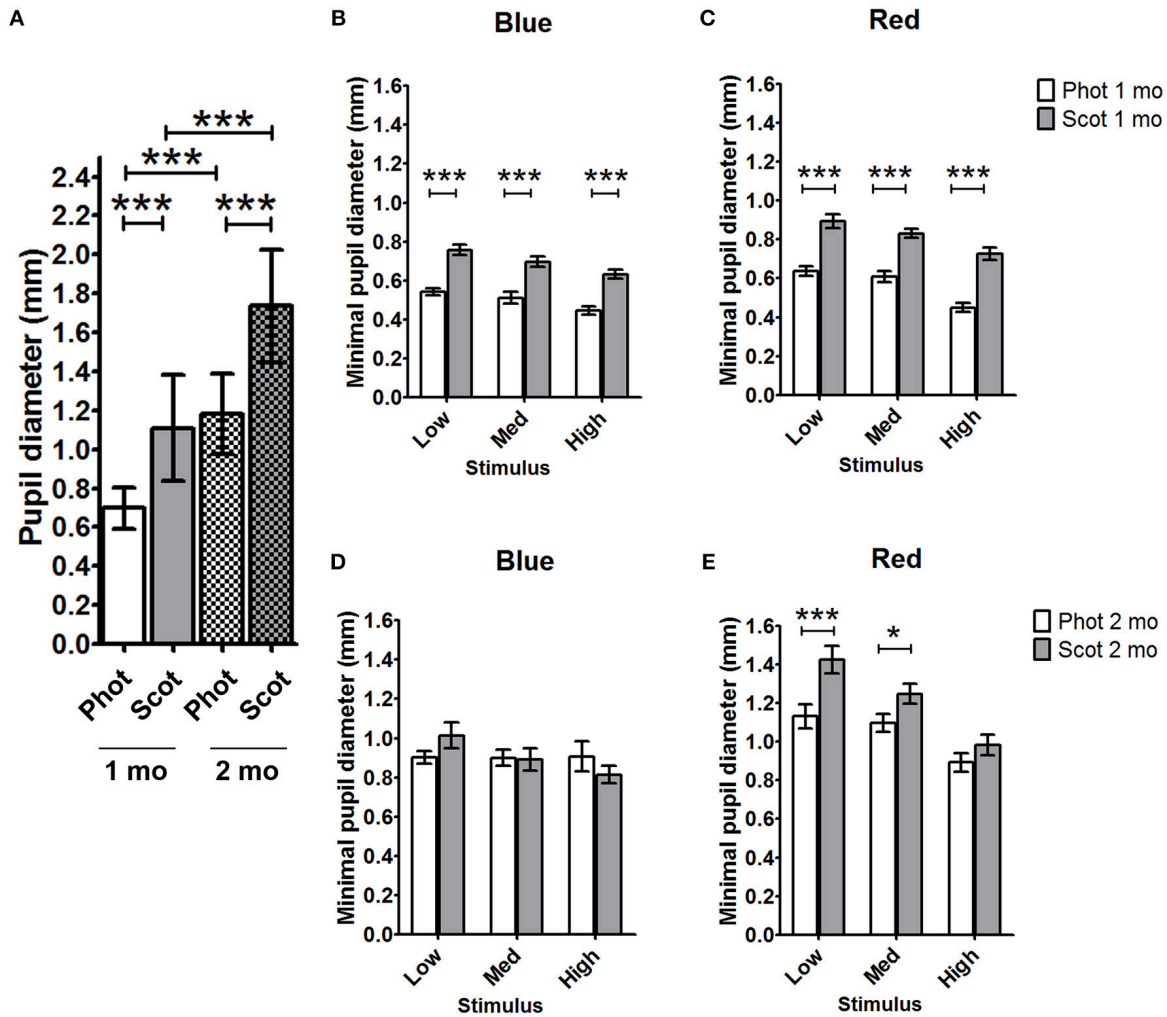
Comparison of C57BL/6 pupil diameters under photopic and scotopic conditions for 1- and 2-month-old animals. **(A)** Baseline pupil diameters for photopic and scotopic conditions in 1- and 2-month-old animals were compared. The minimal pupil diameter for 1-month- **(B,C)** and 2-month- **(D,E)** old mice under scotopic and photopic conditions in response to blue light stimuli **(B,D)** and red-light stimuli **(C,E)** were compared. Phot, photopic conditions; Scot, scotopic conditions; mo, months; Med, medium; ^*^p < 0.05; ^***^p < 0.001.

### Age-Related Changes During the Constriction Phase of the Pupil Response

**Figure 2:**
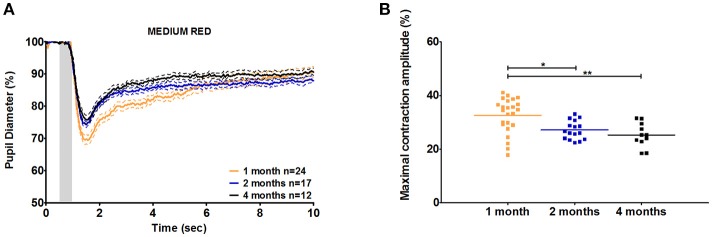
C57BL/6 pupil responses to medium red light in 1-, 2-, and 4-month-old mice. **(A)** Mean pupil diameter (in %) of 1-, 2-, and 4-month-old mice in response to medium red stimulus. The plain lines represent the mean responses of 1-, 2-, and 4-month-old mice and the dotted lines represent the SEM of these means with the same color code. **(B)** Dot plot of the maximal constriction amplitude of 1-, 2-, and 4-month-old mice in response to medium red stimulus. Horizontal lines represent the mean of the different age groups. ^*^p < 0.05; ^**^p < 0.01.

To determine whether age affects the initial pupil constriction in response to light, we compared the maximal constriction amplitude of 1-, 2-, and 4-month-old animals. Except in reaction to medium red stimulus, there were no significant differences in the maximal constriction amplitude between ages. However, 1-month-old C57BL/6 mice showed a greater maximal constriction amplitude in response to medium red stimulus compared to that in 2- and 4-month-old animals (20 and 30% increases, respectively; *p* < 0.01; Figure 2, Table 3, Supplementary Figures 1, 2). This greater constriction amplitude seemed to contrast the smaller pupil diameter of the 1-month-old mice and we subsequently repeated the measurements in another wild-type strain, namely Sv129S6. Similar to that observed with the C57BL/6 strain, 1-month-old Sv129S6 mice showed greater maximal constriction amplitude in response to medium red stimulus. However, unlike the C57BL/6 strain, Sv129S6 mice also showed greater maximal constriction amplitude in response to all other red and all blue stimuli compared to that in 2-month-old mice (Table 4, Supplementary Figures 3, 4).

To better understand the differential pupil response between 1 and 2 months of age, we recorded the PLR in C57BL/6 mice using the same protocol but under photopic conditions. As expected, the baseline pupil diameter was smaller under photopic conditions than under scotopic conditions for both 1- and 2-month-old C57BL/6 animals (Figure 1A, Table 5; 37 and 32% decreases in diameter at 1 and 2 months, respectively; *p* < 0.001). The maximal constriction amplitude was also decreased under photopic conditions compared to that under scotopic conditions at both ages for all stimuli (Table 5; *p* < 0.001). However, age influenced the effect of light conditions on the minimal diameter (at maximal constriction). Whereas for 1-month-old animals the minimal diameter in response to all stimuli was 28–34% smaller under photopic conditions compared to that with scotopic conditions (Figure 1, Table 5; *p* < 0.001), for 2-month-old animals, the minimal diameters in response to high red and all blue stimuli were not significantly different between the two conditions. At 2 months of age, the only significant decrease in minimal diameter under photopic conditions (compared to that under scotopic conditions) occurred in response to low and medium red stimuli (Figure 1, Table 5; 29 and 12% decreases in diameter; *p* < 0.001 and *p* < 0.05, respectively). These results showed that the response of 1-month-old mouse pupils is highly affected by the light conditions (scotopic or photopic), whereas in 2-month-old mice, only responses to low and medium red are modified by photopic conditions.

**Table 5:** Comparison of pupil light response (PLR) under photopic and scotopic conditions in C57BL/6 mice at 1 and 2 months of age.

	**1 month**			**2 months**		
	**Mean**	**SEM**	***n***	**Mean**	**SEM**	***n***
**PHOTOPIC BASELINE PUPIL DIAMETER (mm)**						
	0.70^1^	0.02	19	1.18^*^^1^	0.06	13
**SCOTOPIC BASELINE PUPIL DIAMETER (mm)**						
	1.11	0.07	16	1.74^*^	0.08	15
**PHOTOPIC MAXIMAL CONSTRICTION AMPLITUDE (%)**						
Low blue	20.74^1^	0.99	21	19.77^1^	1.53	13
Medium blue	25.63^1^	1.80	20	26.46^1^	1.40	12
High blue	33.16^1^	1.93	20	29.80^1^	3.11	9
Low red	8.82^1^	1.06	19	4.54^1^	0.873	13
Medium red	14.01^1^	1.49	19	10.51^1^	0.80	15
High red	30.64^1^	1.45	21	28.47^1^	1.69	15
**SCOTOPIC MAXIMAL CONSTRICTION AMPLITUDE (%)**						
Low blue	36.27	1.02	23	36.38	1.34	13
Medium blue	43.86	1.32	23	45.43	2.45	14
High blue	52.42	1.87	16	53.10	1.82	16
Low red	20.19	1.26	22	15.35	1.14	17
Medium red	32.61	1.32	24	27.15^#^	0.86	16
High red	44.96	1.80	20	43.79	1.74	16
**PHOTOPIC MINIMAL DIAMETER (mm)**						
Low blue	0.54^1^	0.02	21	0.90^*^	0.03	13
Medium blue	0.51^1^	0.03	20	0.90^*^	0.03	12
High blue	0.45^1^	0.02	20	0.91^*^	0.08	9
Low red	0.64^1^	0.03	19	1.13^*^^1^	0.06	13
Medium red	0.61^1^	0.03	19	1.10^*^^2^	0.05	15
High red	0.45^1^	0.02	21	0.89^*^	0.05	14
**SCOTOPIC MINIMAL DIAMETER (mm)**						
Low blue	0.76	0.03	23	1.01^*^	0.07	13
Medium blue	0.70	0.03	23	0.89^*^	0.06	14
High blue	0.65	0.03	16	0.81^†^	0.05	15
Low red	0.88	0.04	21	1.43^*^	0.07	17
Medium red	0.84	0.03	25	1.25^*^	0.05	17
High red	0.68	0.03	25	0.98^*^	0.05	16

*Quantifications of the baseline pupil diameter (mm) and the relative maximal constriction amplitude (%) are reported*.

* *Significantly different compared to 1-month-old mice under the same conditions, p < 0.001*;

† *significantly different compared to 1-month-old animals under the same conditions, p < 0.01*;

# *significantly different compared to 1-month-old mice under the same conditions, p < 0.05*.

1 *significantly different compared to scotopic conditions based on mice of the same age, p < 0.001*;

2 *significantly different compared to scotopic conditions based on mice of the same age, p < 0.05*.

**Figure 3:**
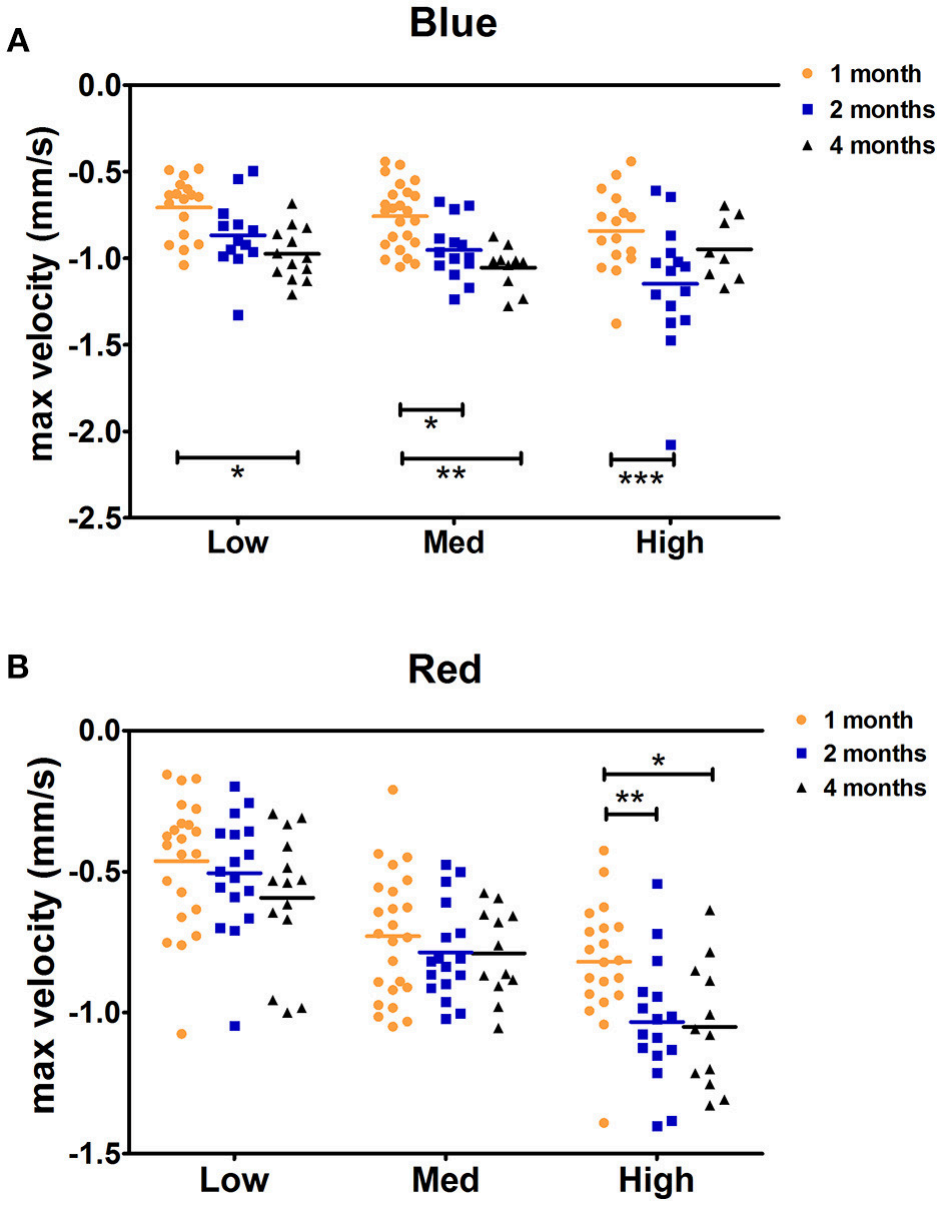
C57BL/6 maximal constriction velocity for 1-, 2-, and 4-month-old animals. Dot plot of the maximal velocity of constriction in response to blue **(A)** and red **(B)** light stimuli is shown. Horizontal lines represent the means of different age groups. ^*^p < 0.05; ^**^p < 0.01; ^***^p < 0.001.

The maximal peak velocity in response to low and medium red stimuli was not significantly different among C57BL/6 mice aged 1, 2, and 4 months. However, in response to high red and low and medium blue, 1-month-old animals exhibited significantly smaller maximal velocity compared to that in 4-month-old animals (Figure 3). Measures for 2-month-old animals followed a trend regarding the age-dependent effect on maximal velocity, but significant differences compared to those in 1-month-old mice were only observed in response to high red and medium blue stimuli. In response to high blue, the 1-month-old maximal velocity was smaller than the 2-month-old maximal velocity but not that of 4-month-old animals. Thus, we showed that during the constriction phase, age can affect both the maximal constriction amplitude and maximal velocity of the pupil response to particular stimuli based on our protocol.

### Age-Related Changes During the Recovery Phase of the Pupil Response

**Figure 4:**
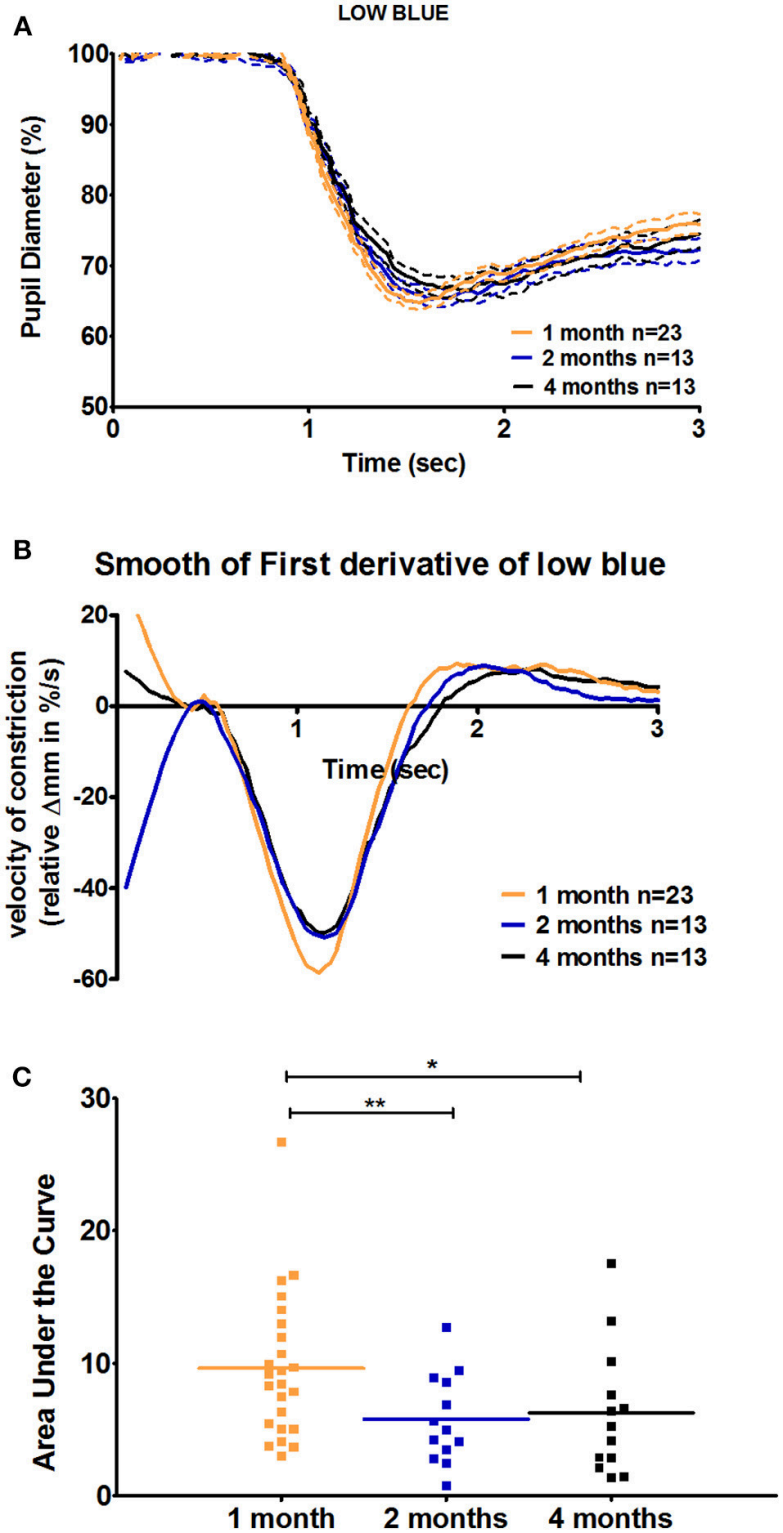
Quantification of the positive peak of the first derivative of the response to low blue stimuli in C57BL/6 mice. **(A)** Magnification of the mean pupil response to low blue stimuli during the first 3 s of the protocol. The plain lines represent the mean responses and dotted lines represent the respective SEMs of these means. **(B)** The smooth first derivative of the pupil response is shown in **(A)**. **(C)** Dot plot of the area under the curve of the positive peak observed after light offset, based on the graph **(B)**. Horizontal lines represent the means of the different age groups. ^*^p < 0.05; ^**^p < 0.01.

For C57BL/6 mice, except in response to low blue stimulus, there was no significant difference between ages with respect to the early partial dilation of the recovery phase in response to all other stimuli. The AUC of the first derivative of the response to low blue was significantly greater in 1-month-old mice compared to that in 2- and 4-month-old mice (66 and 53% increase at 1 month compared to that at 2 and 4 months, *p* < 0.01 and *p* < 0.05, respectively; Figure 4, Table 3). For Sv129S6 mice, significantly larger AUC values were noted for both low and medium red responses at 1 month compared to those at 2 months (Table 4).

**Figure 5:**
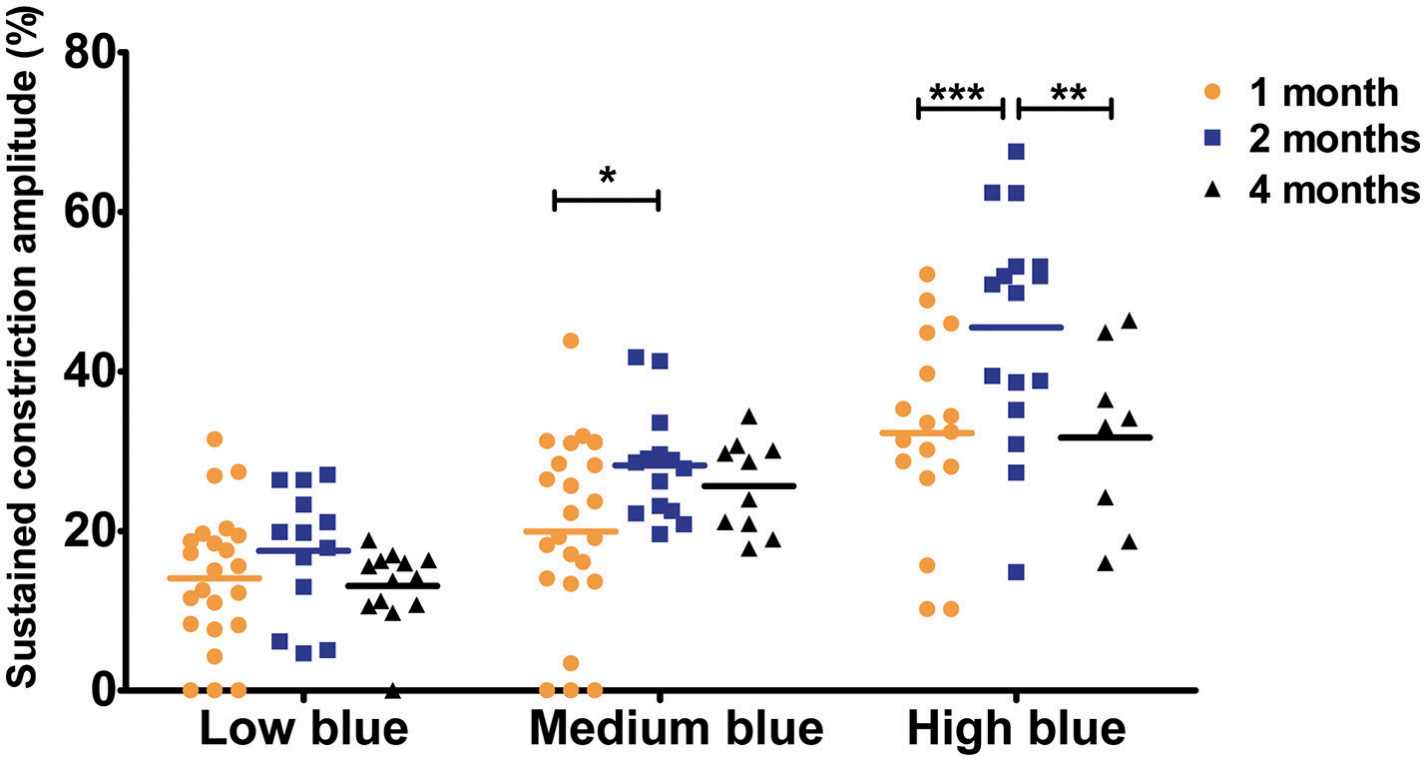
Sustained constriction amplitude for 1-, 2-, and 4-month-old C57BL/6 mice in response to blue stimuli. Dot plot of sustained response after 9.5 s of light offset for 1-, 2-, and 4-month-old mice. One-month old mice exhibited significantly smaller sustained constriction in response to medium and high blue light compared to that in 2-month-old animals. Horizontal lines represent the mean of the different age groups. ^*^p < 0.05; ^**^p < 0.01; ^***^p < 0.001.

To analyze the later phase of recovery, we compared relative pupil diameters 9.5 s after stimuli offset (sustained constriction). The only significant difference between age groups was in response to medium and high blue stimuli. Specifically, 1-month-old C57BL/6 mice a had decreased sustained constriction amplitude in response to these stimuli compared to that in 2-month-old, but not 4-month-old, mice (Figure 5, Table 3, 30% decreased amplitude at 1 month compared to that in 2 months, in response to medium and high blue stimuli, *p* < 0.05 and *p* < 0.001 respectively). A significant decrease was even observed in 4-month-old animals in response to high blue light compared to that in 2-month-old mice (Table 3, *p* < 0.01). Assessing the ratio of sustained constriction amplitude to maximal constriction amplitude gave similar results. No significant differences between ages were observed in terms of the relative recovery in response to all stimuli, except in response to medium blue and high blue light (Table 3, *p* > 0.05). In response to these stimuli, 1-month-old mice exhibited a significantly smaller ratio than 2-month-old, but not 4-month-old, mice. For Sv129S6 mice, no significant differences were found in terms of the sustained response or the ratio of response to all stimuli (Table 4, *p* > 0.05). These results revealed the limited modification of the recovery phase in response to medium and high blue light at 2 months of age in C57BL/6, but not Sv129S6, mice.

### Age-Related Changes in Retinal Activity

**Figure 6:**
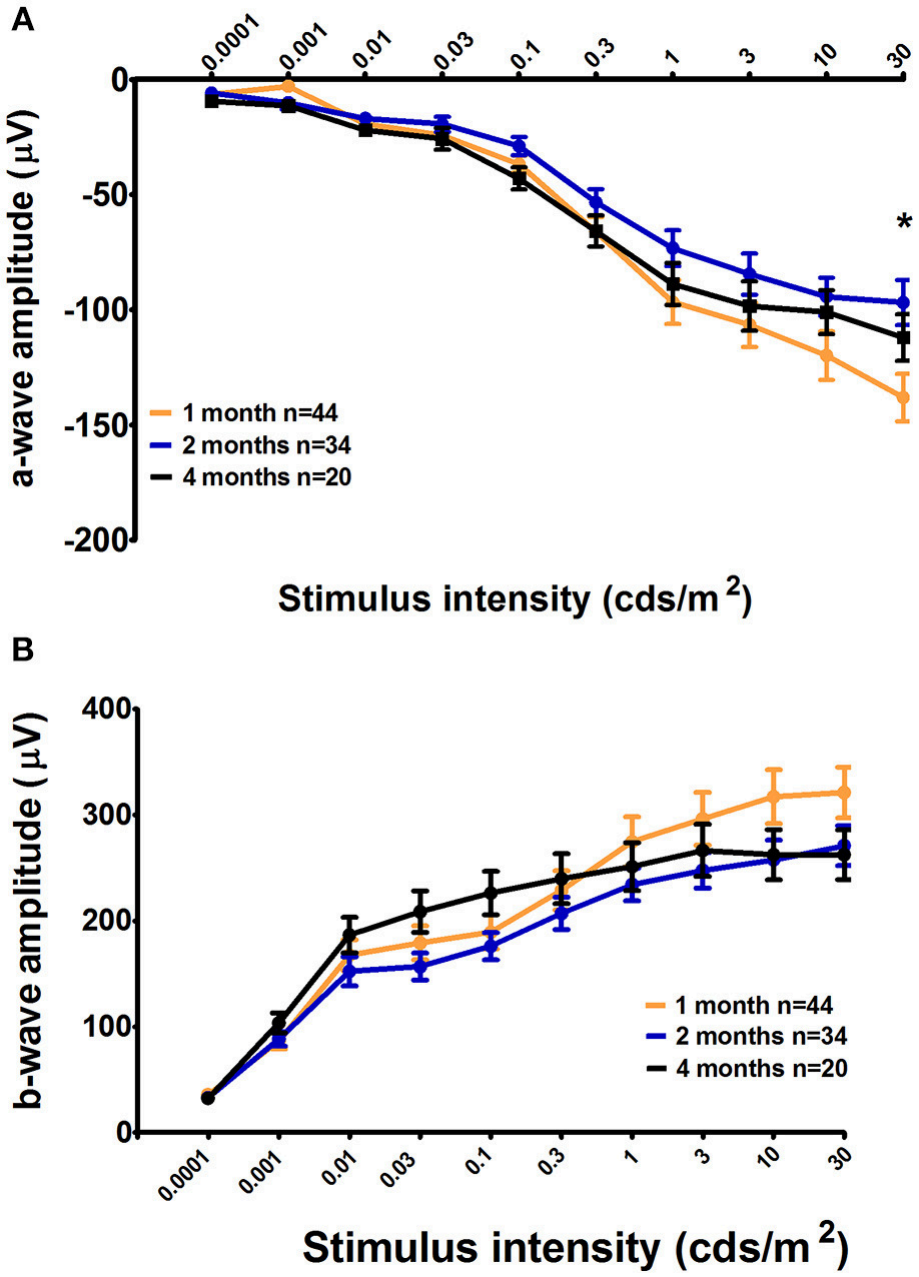
Retinal activity in 1-, 2-, and 4-month-old C57BL/6 mice. The mean a-wave and b-wave amplitudes, with SEM error bars, measured under scotopic conditions, are represented as a function of the stimulus intensities for graph **(A)** and **(B)**, respectively. One-month-old C57BL/6 mice (light gray) displayed a larger maximal retinal response for the a-wave under scotopic conditions as measured by an electroretinogram (ERG) compared to that in 2-month-old mice (medium gray). The 4-month-old animal responses were not significantly different from those at 1 and 2 months. ^*^p < 0.05.

In parallel to PLR recordings, retinal activity was measured by ERG. For C57BL/6 mice, we observed significant increase in the scotopic a-wave amplitude of 1-month-old mice compared to that of 2-month-old animals only in response to the highest stimulus intensity; however, with an intermediate value, the amplitude in 4-month-old mice was not significantly different from that in either 1- or 2-month-old mice (1 month = −138.1 ± 10.27 μV, 2 months = −96.84 ± 9.84 μV, and 4 months = −112.01 ± 10.06; 1 month vs. 2 month, *p* < 0.001; Figure 6A). No other significant differences were found in terms of the ERG parameters among the three age groups in this strain (Figure 6B). Further, no significant age-specific effects on any ERG parameters examined were noted in Sv129S6 mice (Supplementary Figure 5).

### Comparison of Retina Structure Between Mice of 1 and 2 Months of Age

**Figure 7:**
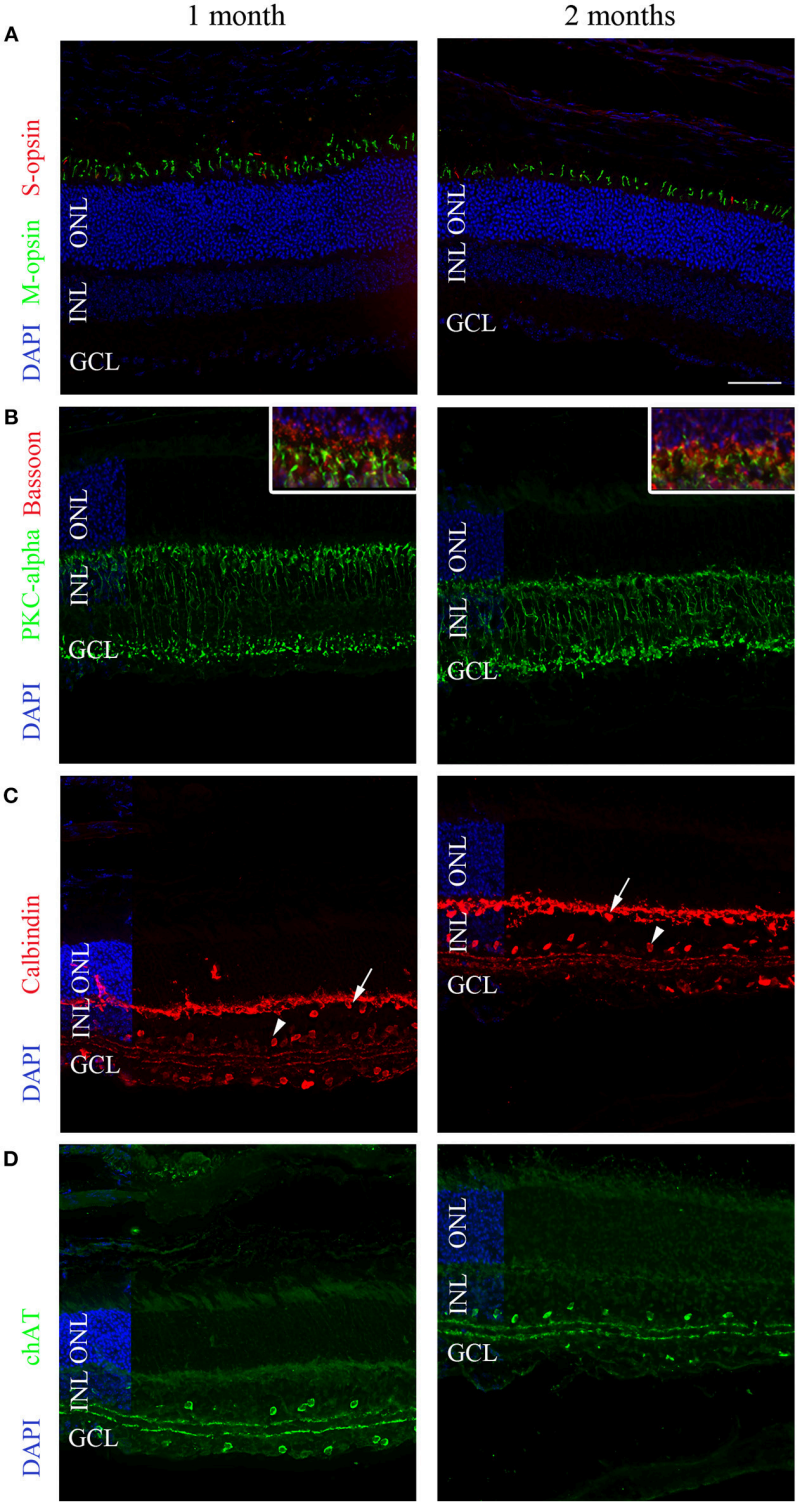
Representative immunolabeling of the retinas of 1- and 2-month-old C57BL/6 mice. **(A)** A region in the superior hemisphere of 1- and 2-month-old retinas showed a similar number of M-cones (green) and S-cones (red) labeled by anti-MWL-opsin and anti-S-opsin antibodies, respectively. **(B)** Bipolar cells labeled with anti-PKCalpha (green) were similar between 1- and 2-month-old retinas. The inset shows 2-fold magnification of B at the photoreceptor termini with co-labeling for Bassoon (red). **(C)** The calbindin labeling of horizontal (arrows), amacrine (arrowheads), displaced amacrine, and ganglion cells was similar at 1 and 2 months. **(D)** The labeling of cholinergic amacrine cells using an anti-ChAT antibody was similar at 1 and 2 months. DAPI (blue) was used as a counterstain for panels **(A–D)**. The horizontal line in **(A)** represents 50 μm for all panels and 25 μm for the inset of **(B)**. ONL, outer nuclear layer; INL, inner nuclear layer; GCL; ganglion cell layer.

The origin of the modified pupil response between 1 and 2 months of age was unknown, but one possibility was suggested to be changes in retinal structure, particularly in the outer layer. We thus evaluated the histopathology of the retina in these two age groups. Specifically, we labeled cones, rods, bipolar cells, horizontal cells, and amacrine cells with different antibodies and analyzed the central section bisecting the optic nerve of each eye (*n* = 3 for each group; Figure 7).

Cone photoreceptors were labeled with anti-S-opsin and anti-MWL-opsin antibodies; no significant difference in the number of positive cells was observed. In the central section, an average of 573 ± 91.25 S-opsin-positive cells were counted for 1-month-old animals, whereas 472 ± 21 positive cells were observed for 2-month-old mice (*p* > 0.05). For MWL-opsin, 822 ± 133 and 710 ± 14 positive cells were counted for 1- and 2-month-old animals, respectively (*p* > 0.05). To study rod photoreceptors, we labeled the outer segment with an antibody directed against the rod transducing GNAT1 protein. No differences in labeling intensity in the outer segments were found between 1- and 2-month-old mice. The thickness of the photoreceptor layers was also similar between both age groups. Rod bipolar cells were then analyzed using the marker PKC-alpha. No differences in the number of PKC-alpha-positive cells were observed (638 ± 50 at 1 month and 650 ± 26 at 2 months of age; *p* > 0.05). For the presynaptic nerve terminals labeled with an anti-bassoon antibody, no obvious changes in intensity or amount of staining were noted between 1- and 2-month-old animals.

Next, we used an anti-calbindin antibody to quantify horizontal cells and amacrine cells in the inner nuclear layer and displaced amacrine cells in the ganglion cell layer, in addition to ganglion cells. No difference was observed in terms of horizontal cells (96 ± 25 at 1 month and 94 ± 3.2 at 2 months of age), amacrine cells (373 ± 44 at 1 month and 268 ± 21 at 2 months of age), or displaced amacrine cells and ganglion cells (163 ± 18.5 at 1 month and 141 ± 9.8 at 2 months of age).

Finally, an anti-ChAT antibody was used to label cholinergic amacrine cells. No differences in the number of positive cells were noted (140 ± 0 vs. 100 ± 81 at 1 and 2 months of age, respectively).

## Discussion

The comparison of PLR and ERG measurements at different ages revealed alterations in pupil and retinal responses that occurred with age. Specifically, 1-month-old animals clearly showed different features compared to older animals. Currently, we cannot identify the mechanisms underlying these age-related changes, but several explanations can be proposed, such as morphological differences, the maturation of the iris sphincter, changes in retinal sensitivity, or refined central control of the pupil. These hypotheses will be considered in relation to the significant results discussed as follows.

The smaller baseline diameters (in scotopic and photopic conditions) of 1-month-old animals (36% decrease compared to that in 2-month-old C57BL/6 mice) cannot be entirely explained by morphological size differences between 1- and 2-month-old groups because the eye cup diameter was found to only be decreased by 4% in 1-month-old animals (personal unpublished data). Furthermore, morphological differences would not explain the increase in maximal constriction amplitude in response to particular photoreceptor stimuli at 1 month of age because the relative quantification of baseline parameters took into account the starting diameter.

Another explanation for these age-related differences could be the maturation of the iris sphincter. The smaller baseline pupil diameter and increased maximal constriction amplitudes could result from an immature and stronger iris at 1 month of age. However, the slower maximal velocity and faster recovery suggest decreased iris sphincter efficacy at this age. Additionally, variations in these PLR features were observed only following specific stimuli, which is not consistent with general iris immaturity that would affect all responses similarly. For these reasons, maturation of the iris sphincter is probably not the origin of PLR variations observed with age.

Several of the PLR metrics analyzed in our study suggest higher retinal sensitivity at 1 month of age compared to that in older mice. First, the baseline diameter after dark adaptation was smaller at 1 month of age for both strains. The same difference was noticed under photopic conditions for 1-month-old C57BL/6 mice. Second, the maximal constriction amplitudes in response to medium (C57BL/6) or low and medium (Sv129S6) red stimuli were larger at 1 month of age. Considering that these stimuli were previously shown to be rod- and cone-driven in mice (13), these results suggest an increase in rod and cone input in 1-month-old mice. Likewise, in C57BL/6 mice, the ERG response to scotopic conditions was associated with a larger maximal mixed cone–rod response at this age, demonstrating that 1-month-old animals exhibit heightened response to light. Of note, in Sv129S6 mice, we could not confirm the increased maximal cone–rod response. Moreover, in this genetic background, the maximal constriction amplitude was greater in response to all other stimuli at 1 month of age. This latter result suggests that in this strain, a major change in the PLR process occurs between 1 and 2 months of age, which might not be directly linked to rods and cones, but rather to variation in pathways that control the entire pupil. At this stage of the study, we cannot distinguish between changes in peripheral or central pathways. Natural variances exist between wild-type mouse strains, which could explain the differences between C57BL/6 and Sv129S6 mice observed in our study. Previously, different neurochemical profiles have been highlighted, which could account for different behavioral responses between strains (29). For example, basal levels of ionotropic glutamate receptor subunit vary according to mouse strain (30). Such diversity might also be present in the neural retina and could induce small deviations in terms of retinal visual processing among non-pathological mouse strains. A variation in the course of retinal degeneration was noted between C57BL/6 and Sv129S6 mice when examining the effect of rhodopsin knockouts on the retina (28). Genetic modifiers were proposed to modulate the survival of photoreceptors in these models and could also affect neural development processes, which might account for the dissimilarity of pupillary and ERG responses observed between these strains.

An additional hypothesis for the mechanism responsible for age-related changes in the PLR is that the pupil circuitry, associated with all three types of photosensitive cells, is adjusted after 1 month. Since melanopsin cells were previously shown to mediate the steady state of the pupil, these cells could be implicated in the reduced baseline pupil size observed at 1 month. Recording the PLR under photopic conditions allows for the examination of potential changes in retina circuitry that have been implicated in adaptation between 1 and 2 months of age (16, 17). As expected, because of the adaptation to background light and the subsequent smaller baseline diameter, the maximal constriction amplitude (in %) in C57BL/6 mice was reduced under photopic conditions compared to that under scotopic conditions. More importantly, age was found to influence the effect of photopic conditions on the minimal diameter (in mm). Whereas at 1 month of age, in response to all stimuli, the minimal diameter was smaller under photopic conditions, at 2 months of age, this only occurred in response to low and medium red light (rod and cone-driven stimuli). Thus, in response to the specific rod- and cone-driven stimuli, photopic conditions induce a decrease in the minimal diameter independent of age. However, in response to all other stimuli (also directly implicating melanopsin cells), photopic conditions promoted a decrease in the minimal diameter only in 1-month-old animals. In this case, the smaller diameter could indicate improper integration of background light, which would result in some type of additive process comprising rod and cone input and melanopsin input. This experiment revealed the immature control of the PLR under photopic conditions at 1 month of age in C57BL/6 mice; however, we did not perform similar photopic examinations using Sv129S6 mice to confirm this result. Replicating such experiments in this strain could determine if this change in photopic sensitivity between 1 and 2 months is common to both wild-type strains.

In the early phase of recovery, the more pronounced early partial dilation observed in younger animals is a precise characteristic of rod and cone inputs (13). The setup of early recovery control thus also occurs between 1-month-old and older-aged mice. How the rod and cone inputs, which are transient and linked to light onset, play a role in the recovery phase is not well understood. In 1-month-old C57BL/6 mice, faster recovery was also observed when measuring the sustained amplitude at 9.5 s in response to medium and high blue light, two stimuli expected to be biased toward melanopsin input (13, 16). In humans, Adhikari et al. (12) reported the contribution of rhodopsin and melanopsin to the early recovery phase when subjects were pre-adapted to light. They showed that during the 1.7 s after stimulus offset, both rods and melanopsin were implicated in the early phase of recovery, whereas after 1.7 s post-stimulus offset, dilation was mainly controlled by melanopsin. The faster early and late recovery described in this study at 1 month of age is in accordance with the incomplete maturation of rod- and melanopsin-driven circuitry.

The changes in the pupil response at 1 month of age could reveal the functional refinement of photoreceptor (rod, cone, and/or melanopsin cells) input between 1 and 2 months of age. This hypothesis is consistent with the ERG results obtained from C57BL/6 mice and with the literature wherein most studies showed that ERG measures of retinal response differ between young animals after eye opening and adult mice, and increases until 1 month of age (19, 31). More importantly, in rats, Chaychi et al. (21) showed that the ERG response decreases with age between 1 and 2 months of age (21), consistently with results described in this study. In C57BL/6 mice, Vistamehr and Tian (32) observed the same decrease in a- and b-wave amplitudes from P30 to P60, but this effect did not reach significance (32). Nevertheless, in this study, oscillatory potential amplitudes were significantly reduced from P30 to P60 and to P90. Since oscillatory potential reflects the interaction between bipolar, amacrine cells and retinal ganglion cells, this finding could also reflect the refinement of the retinal circuitry for the PLR.

Whereas our results suggest modifications of the PLR circuitry that occur with age, our histological data did not reveal obvious changes in retinal composition between 1- and 2-month-old mice for classical rod, cone, amacrine, horizontal, and ganglion cells. However, we cannot exclude subtle changes in connections between cells at the outer or inner plexiform layer, as well as in the afferent and efferent pathways involved in the PLR. Further work using whole-mount techniques and electron microscopy is essential to reveal cellular morphological changes and are needed to define the biological basis of PLR refinement between 1 and 2 months of age. Nevertheless, this report shows that in mice, age affects both transient and steady-state mouse pupil diameters. Our results suggest that functional maturation of the retina still takes place after 1 month of age, indicating that studies on adult mouse retinal function should be performed on animals 2 months of age or older. This work also emphasizes the need for the use of adequate control animals of the same background age when PLR is used to explore retinal dystrophy models.

## Author Contributions

CK and NK conceived, designed, and supervised the project. NK, CM, and SC performed the experiments. NK, SC, AK, and CK participated in the analysis and interpretation of the data. NK and CK wrote the manuscript. All authors critically revised and approved the final version of the manuscript.

### Conflict of Interest Statement

The authors declare that the research was conducted in the absence of any commercial or financial relationships that could be construed as a potential conflict of interest.
