# Supplementary material for: Maturation of the Pupil Light Reflex Occurs Until Adulthood in Mice
Source: Front Neurol. 2019 Feb 4;10:56. doi: 10.3389/fneur.2019.00056 (PMC6369172; doi:10.3389/fneur.2019.00056)
Supplement: Supplementary file 1 [file Data_Sheet_1.docx]

Supplementary Material

Maturation of the pupil light reflex occurs until adulthood in mice

**Noémie Kircher, Sylvain V.Crippa, Catherine Martin, Aki Kawasaki and Corinne Kostic***

*** Correspondence:** Corresponding Author: [corinne.kostic@fa2.ch](mailto:corinne.kostic@fa2.ch)


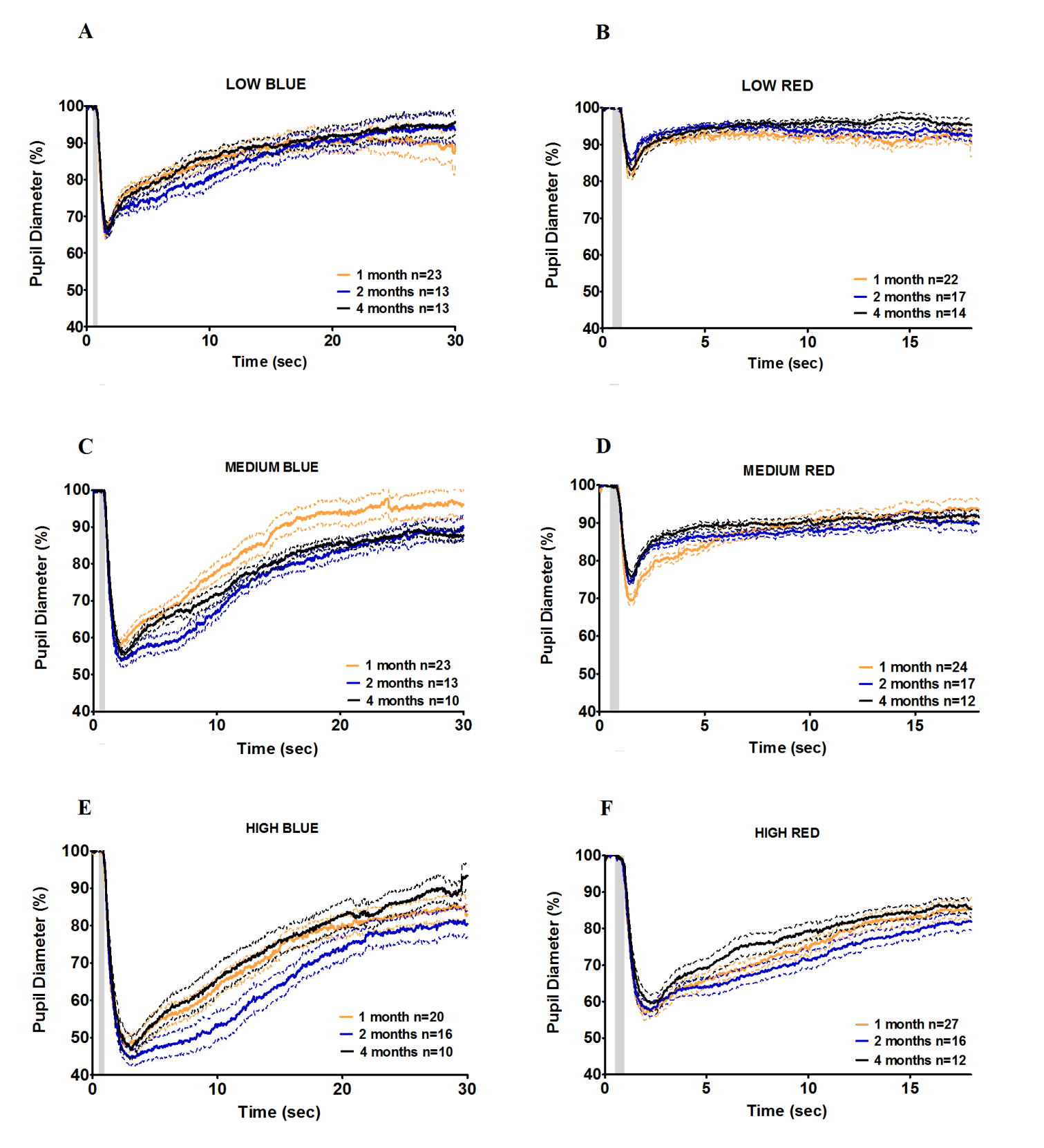


**Supplementary Figure 1.** C57BL/6 pupil responses for 1-, 2-, and 4-month-old mice. Mean pupil diameter (%) of 1-,2-, and 4-month-old mice (plain lines) in response to blue (A,C,E) and red (B,D,F) light. The dotted lines represent the SEM of these means with the same color code.


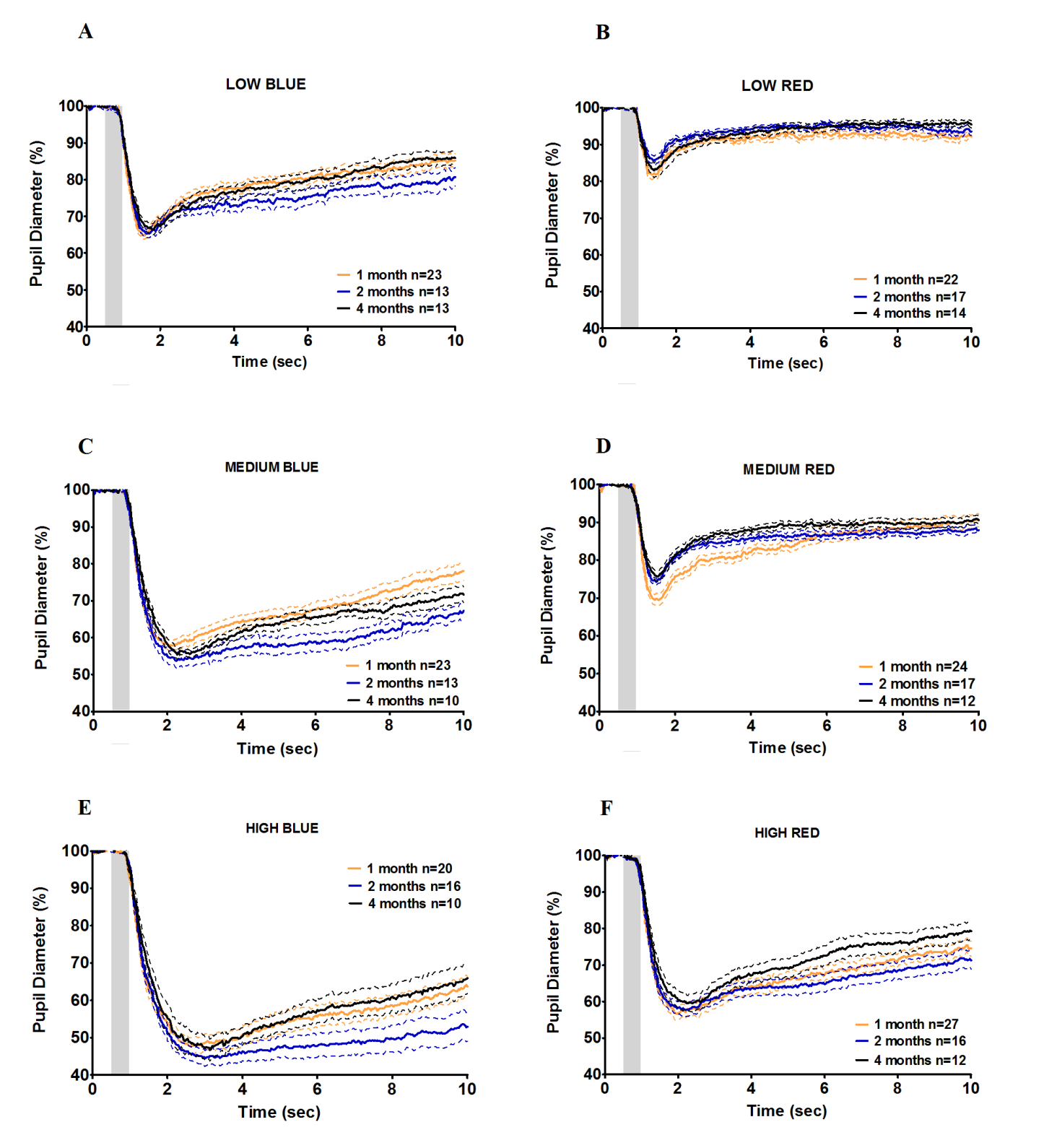
**Supplementary Figure 2.** The ten first seconds of C57BL/6 pupil responses for 1-, 2-, and 4-month-old mice. Mean pupil diameter (%) of 1-,2-, and 4-month-old mice (plain lines) in response to blue (A,C,E) and red (B,D,F) light. The dotted lines represent the SEM of these means with the same color code.


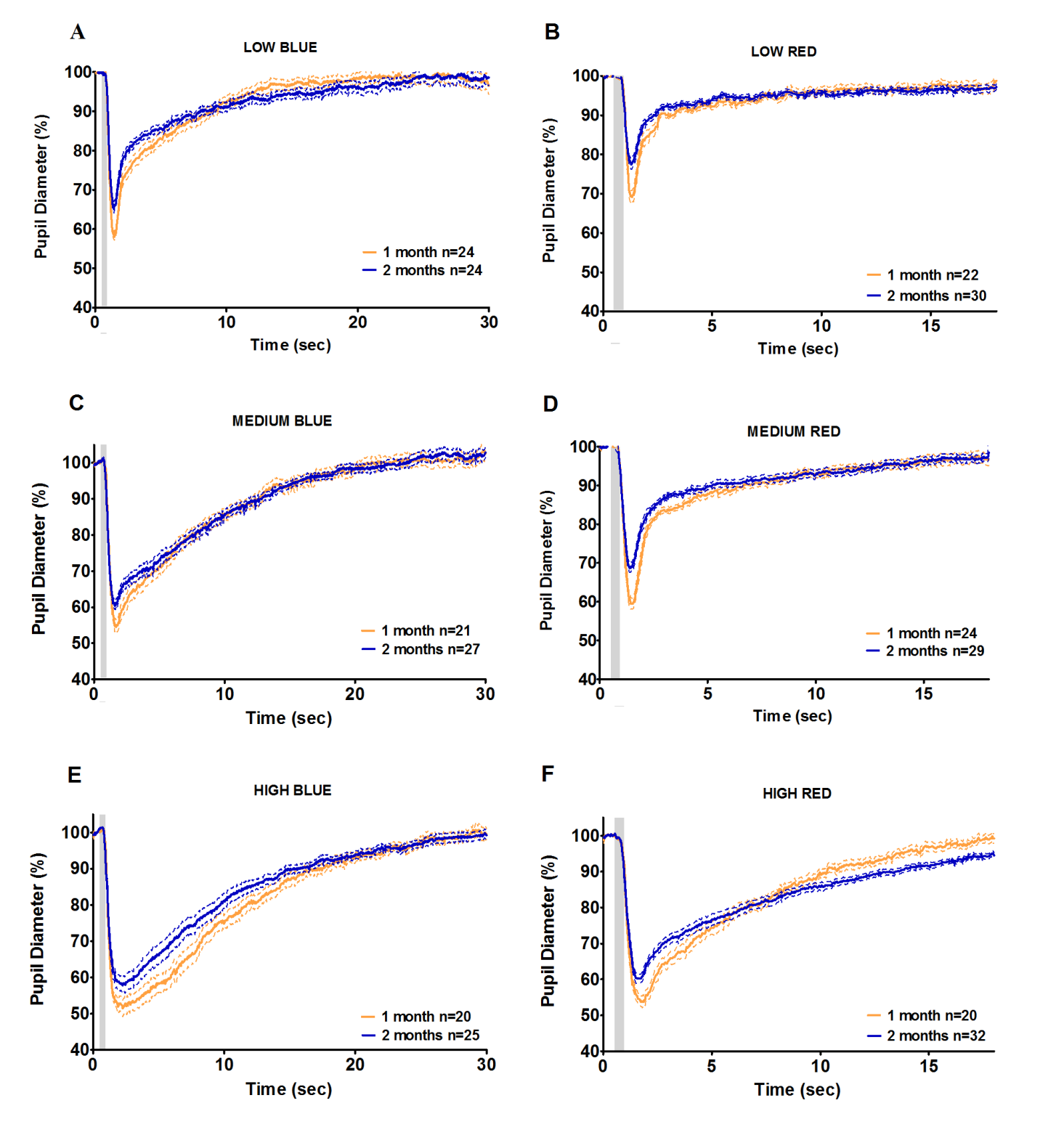


**Supplementary Figure 3.** Sv129S6 pupil responses for 1- and 2-month-old mice. Mean pupil diameter (in %) of 1- and 2-month-old mice (plain lines) in response to blue (A,C,E) and red (B,D,F) light. The dotted lines represent the SEM of these means with the same color code.

**
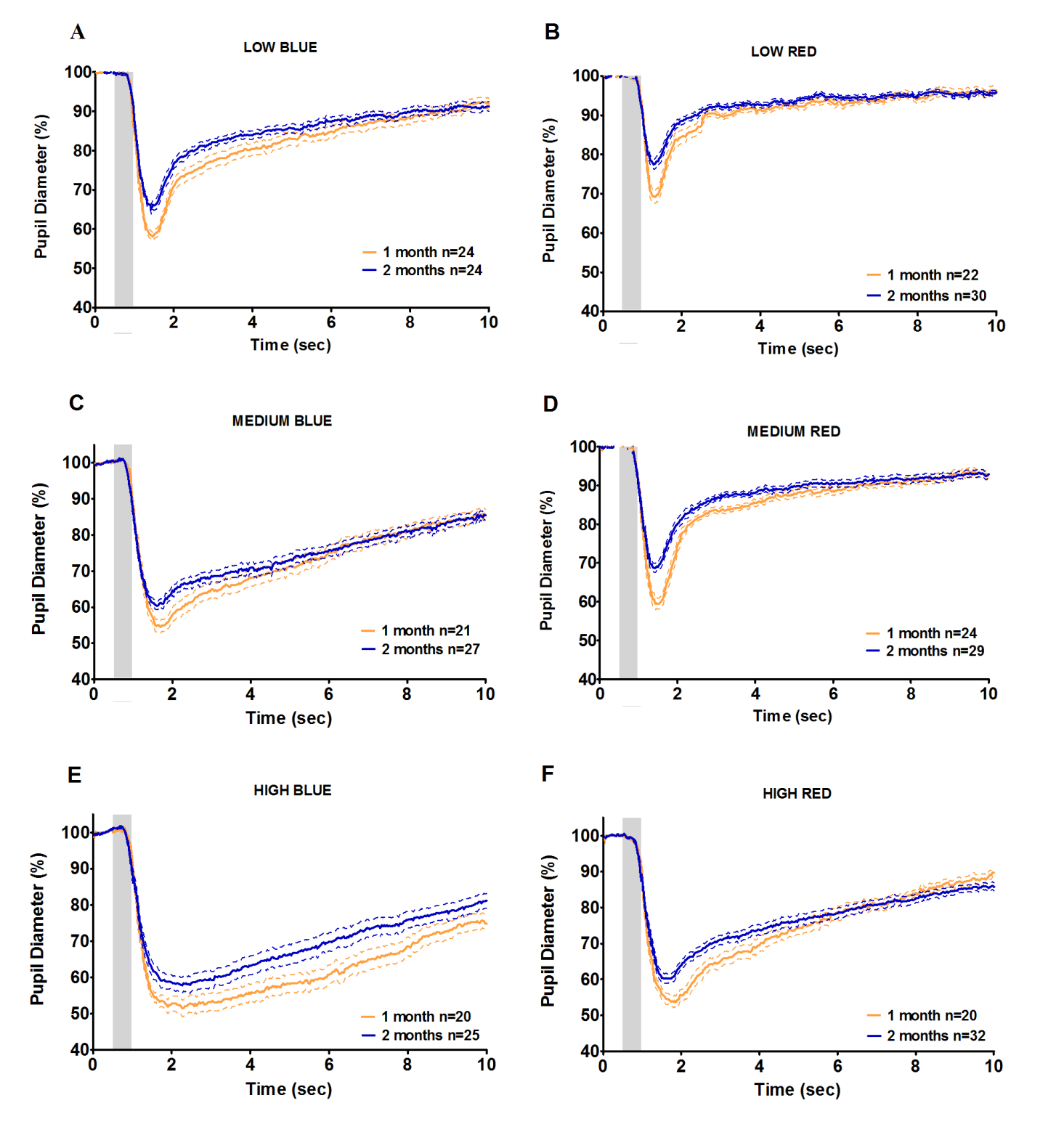
**

**Supplementary Figure 4.** **.** The ten first seconds of Sv129S pupil responses for 1- and 2-month-old mice. Mean pupil diameter (in %) of 1- and 2-month-old mice (plain lines) in response to blue (A,C,E) and red (B,D,F) light. The dotted lines represent the SEM of these means with the same color code.


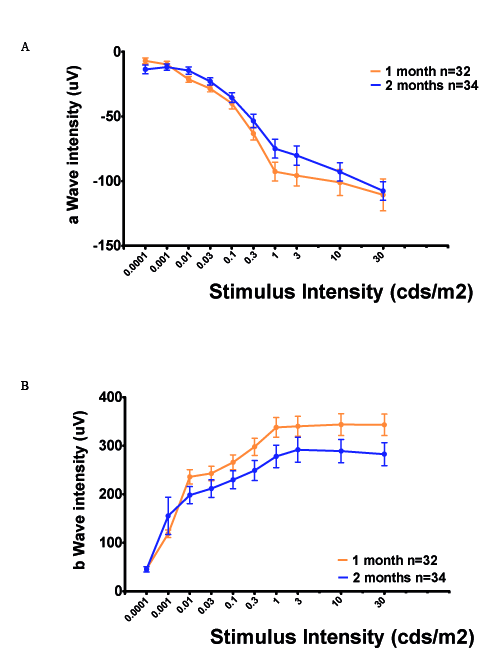


**Supplementary Figure 5.** Retinal activity in 1-, and 2-month-old Sv129S6 mice. The mean a-wave and b-wave amplitudes, with SEM error bars, measured under scotopic conditions, are represented as a function of the stimulus intensities for graph (A) and (B), respectively.
